# Cross-sectional survey on independent mobility of people with dementia: a caregivers’ perspective

**DOI:** 10.1590/1980-5764-DN-2025-0284

**Published:** 2025-09-19

**Authors:** Ise Anderson Orobor, Ramy Hammady, Mary Kennedy

**Affiliations:** 1 University of Essex, School of Computer Science and Electronic Engineering, Colchester Essex, United Kingdom.; 2 Federal University of Petroleum Resources, College of Computing, Department of Computer Science, Effurun Delta State, Nigeria.; 3 University of Southampton, Winchester School of Art, Winchester, United Kingdom.; 4 Helwan University, Faculty of Applied Arts, Dokki Giza, Egypt.; 5 University of Essex, School of Health and Social Care, Colchester Essex, United Kingdom.

**Keywords:** Dementia, Home Environment, Cognitive Dysfunction, Caregivers, Safety, Adult, Demência, Ambiente Domiciliar, Disfunção Cognitiva, Cuidadores, Segurança, Adulto

## Abstract

Dementia significantly impairs cognitive function and severely affects daily living activities. To support independent mobility in older adults and individuals with dementia, home modification strategies, such as safety adaptations, have been identified as critical interventions.

**Objective:**

To explore caregivers’ perspectives on the potentials of digital interventions in enhancing independent mobility for PwD in mild to moderate stages of the condition. The aim is to determine if digital intervention could help PwD to effectively use existing home safety interventions and to safely move around their environment.

**Methods:**

A cross-sectional survey was used to gather insights from 121 professional caregivers and family members providing care for PwD. Participants aged 18 years and above were eligible for inclusion. Responses were analysed using R software, employing descriptive statistics, contingency tables, and graphical charts. χ^2^ tests (p<0.05) assessed the relationships between categorical variables, with Cramér’s V measuring association strength (weak relationship if ≤0.30). Cronbach’s alpha demonstrated reliability for mobility factors (0.87, 95%CI 0.810–0.908).

**Results:**

The study revealed that PwD made limited use of existing home safety interventions, with statistically significant findings (p<0.05) across the four mobility factors evaluated. This indicates that the effectiveness of these interventions could be undermined particularly for individuals living alone.

**Conclusion:**

The study found that digital interventions can support PwD in using existing home safety interventions and navigating their environments more independently. It could help the target population know when and how to these interventions thereby increasing the overall goal of their implementations.

## RESUMO.

A demência compromete significativamente a função cognitiva e afeta de forma severa as atividades da vida diária. Para apoiar a mobilidade independente de idosos e pessoas com demência (PcD), estratégias de modificação do domicílio, como adaptações de segurança, têm sido identificadas como intervenções essenciais.

**Objetivo:**

Explorar as perspectivas de cuidadores sobre o potencial de intervenções digitais na promoção da mobilidade independente de PcD nos estágios leve a moderado da condição. O objetivo é determinar se intervenções digitais podem ajudar PcD a utilizarem de forma eficaz as adaptações de segurança já existentes em casa e a se locomoverem com mais segurança no ambiente domiciliar.

**Métodos:**

Foi utilizado um estudo transversal para obter percepções de 121 cuidadores familiares e profissionais que prestam cuidados a PcD. Foram incluídos participantes com 18 anos ou mais. As respostas foram analisadas com o software R, utilizando estatísticas descritivas, tabelas de contingência e gráficos. Testes do χ^2^ (p<0,05) avaliaram as relações entre variáveis categóricas, com o V de Cramér mensurando a força das associações (relação fraca se ≤0,30). A confiabilidade dos fatores de mobilidade foi demonstrada por meio do alfa de Cronbach (0,87; IC95% 0,810–0,908).

**Resultados:**

O estudo revelou que PcD fazem uso limitado das intervenções de segurança doméstica já existentes, com resultados estatisticamente significativos (p<0,05) nos quatro fatores de mobilidade avaliados. Isso indica que a efetividade dessas intervenções pode ser comprometida, especialmente entre aqueles que vivem sozinhos.

**Conclusão:**

O estudo identificou que intervenções digitais podem apoiar PcD no uso adequado de intervenções de segurança já implementadas no domicílio, além de favorecer uma navegação mais independente pelo ambiente. Tais intervenções podem ajudar esse público a saber quando e como utilizar os recursos disponíveis, aumentando a efetividade e o alcance dos objetivos propostos com sua implementação.

## INTRODUCTION

 Dementia is a significant cognitive disorder that deeply impacts activities of daily living^1^. Early-stage symptoms include memory difficulties, challenges with concentration, planning, and organization, misinterpretation of visual information, confusion about time or location, difficulty with thinking, trouble identifying and avoiding objects on the floor^2-4^. Dementia affects individuals differently between the early stages through the end of life^5^ and can lead to a gradual decline in mobility^6^. Mobility encompasses the ability to move from one position to another, such as sitting, standing, transitioning between postures, and walking^7,8^. Strategies to help People with Dementia (PwD) maintain independent mobility include the use of equipment^7^, home modifications^9,10^, and exercise^11^, among others. 

 PwD face a significantly higher annual fall risk of 60-80%, which is double that of cognitively healthy older adults^12^. While mobility aids can provide support and confidence, their use increases the risk of falls threefold for PwD^13,14^. Fall risk arises from a complex combination of physical and environmental factors^15^. Traditional fall prevention strategies that work for older adults without cognitive impairments have proven ineffective for PwD^16^. 

 Studies on independent mobility in older adults and PwD have identified the adoption of home modification strategies^17-19^. Home modifications involve altering living spaces to improve usability, safety, security, and independence for residents^17,20^. Common recommendations include removing rugs/mats, improving lighting (including sensor lighting) and step edge contrast, and installing grab bars, handrails, and wider doorways^4,9,18,21^. The effectiveness of strategies for independent living relies heavily on adherence to recommendations^15^. Despite environments being designed for independence, older adults often struggle to live independently^22^. Caregivers play a critical role in reminding individuals with dementia to use mobility aids consistently^23^; however, many PwD live alone^24,25^, limiting caregiver support. 

 This study, therefore, explored caregivers’ perspectives on the potentials of digital interventions to enhance independent mobility for PwD in the mild to moderate stages of the condition. The aim was to determine whether digital intervention could support PwD in effectively using existing home safety measures and in safely navigating their environment. The remainder of the study is structured as follows: Section 2 details the study methodology; Section 3 presents the quantitative results; Section 4 discusses the findings, including the study’s strengths and limitations; and Section 5 concludes the study. 

## METHODS

### Study design

 This quantitative study employed a cross-sectional survey to gather insights from professional caregivers or family members providing care or support for PwD. The survey materials were developed in accordance with the University of Essex guidelines for ethical approval of research involving human participants^26^. A total of 25 questions were created and subdivided into four sections: Introduction, Caregivers’ Demographics, Safe Mobility Assessment of PwD, and Caregivers’ Perception of Digital Intervention and Readiness. Completeness checks were implemented to ensure that all mandatory questions were answered before participants could proceed to the next survey page. Participants were given the option to review and revise their responses prior to submitting the survey. No identifiable information was collected. 

### Study population and recruitment

 Participants aged 18 years and above were eligible for the study. Eligible participants were either professional caregivers or family members/friends with at least one year of experience in providing care or support for PwD. Recruitment was conducted by posting study information in healthcare workers’ WhatsApp groups and through word-of-mouth. Recruitment took place between February 2024 and April 2024 across England, United Kingdom. Interested individuals were directed to a Google Form to complete the online survey (https://tinyurl.com/yxmh5mpj). Participants were provided with information about the research, along with a separate link to the consent and participant information sheet, prior to accessing the questionnaire. The survey was designed to take approximately 10 minutes and was entirely anonymous and voluntary. The study received ethics approval from the University of Essex Ethics Sub-Committee 2 (Reference numbers: ETH23240711). Submission of the online questionnaire was taken as implied consent for all participants. 

### Measures

Independent mobility of PwD was assessed using five questions: (Q1) availability of home safety intervention; (Q2) awareness of surroundings; (Q3) ability to navigate without assistance; (Q4) ability to recognize when to use home safety interventions without reminders; and (Q5) ability to utilize home safety interventions without being guided. Response options covered three key conditions: 1=Yes, 2=Sometimes, and 3=No.

### Data analysis

 Study responses were analyzed using the R statistical software package. Descriptive statistics were used to compute and summarize participants’ responses through contingency table and graphical chart representation. These techniques provided clearer insight into the distribution, central tendency, and variability of the data. The χ^2^ test^27^ was employed to determine whether a relationship existed between categorical variables, using a significance level α, derived from a threshold of 5% (α=0.05) and a p<0.05. Based on this: 

 If p<0.05 → *H0* is unlikely; the null hypothesis is rejected. Otherwise, if p≥0.05 → *H0* is likely; the null hypothesis is not rejected. 

 Cramér’s V was used to measure the strength of association between variables, with scores≤0.30 indicating a weak relationship^28^. Cronbach’s alpha assessed the reliability of items measuring mobility factors, yielding an overall value of 0.87 with a 95% confidence interval [0.810–0.908]. This result indicates good internal consistency based on Cronbach’s standards^29^. 

 The following hypotheses were tested in the study:

- Null hypothesis (H_0_): Digital interventions cannot help PwD effectively use existing home safety interventions and safely move around their environment independently;
- Alternative hypothesis (H*1*): Digital interventions can help PwD effectively use existing home safety interventions and safely move around their environment independently.

## RESULTS

### Demographic characteristics

 A total of 121 participants were involved in the study. The participants were predominantly female (n=80, 66.12%), aged 30–39 years (n=67, 55.37%), and of African ethnicity (n=82, 67.76%). Majority were healthcare assistants/caregivers (n=91, 75.21%) and provide care or support to PwD in residential or nursing care facilities (n=99, 81.81%). Table 1 presents a comprehensive distribution of participant demographics. 

**Table 1:** Summary of participants’ demographic characteristics.

Participants demographic characteristic (N=121)		N	(%)
Age Range (Years)	18-29	4	3.31
	30-39	67	55.37
	40-49	47	38.84
	50+	3	2.48
Gender	Male	41	33.88
	Female	80	66.12
	Other	0	0
	Prefer not to say	0	0
Ethnicity	White/White British	23	19
	Black/Black British	6	4.95
	Asian/Asian British	8	6.61
	African	82	67.76
	Other	2	1.65
Occupation	Healthcare Assistant/ Caregiver	91	75.21
	Other	30	24.79
Category	Professional	79	65.29
	Family member/Friend	42	34.71
Experience (Years)	1-3	53	43.80
	4-7	60	49.59
	8-11	5	4.13
	12+	3	2.48
Place of care or support	Residential or Nursing care facility	99	81.81
	Domiciliary care	22	18.18
Number of people with dementia cared for or supported	1-3	56	46.28
	4-6	36	29.75
	7-9	8	6.61
	10+	21	17.35
Daily hours spent with people with dementia	1-8	43	35.54
	9-16	38	31.40
	17-24	40	33.06

**Figure 1:**
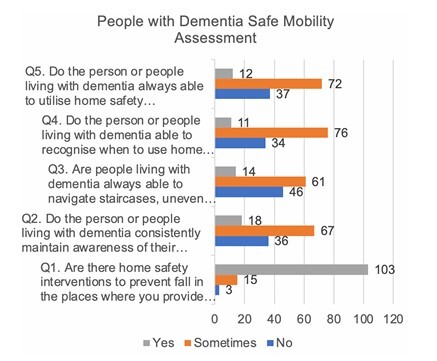
Participants response on question regarding people with dementia safe mobility (N=121).

Table 2 present participants’ responses on the independent mobility of the individuals they care for, as well as their perceptions of digital interventions in dementia care. These data are visualized in a bar chart in Figure 1. 

**Table 2:** Summary of participant response on independent mobility assessment of people with dementia and digital intervention and readiness.

Participant response on independent mobility assessment of people with dementia (N=121)			
Questions	Responses		
	Yes N (%)	Sometimes N (%)	No N (%)
Are there home safety interventions where you provide care or support to aid people with dementia?	103 (85.12)	15 (12.40)	3 (2.48)
Do individuals living with dementia consistently maintain awareness of their surroundings, avoiding collisions with objects or an unsafe environment?	18 (14.88)	67 (55.37)	36 (29.75)
Assuming there is no physical mobility impairment, are people living with dementia always able to navigate staircases, uneven surfaces, and obstacles safely without assistance?	14 (11.57)	61 (50.41)	46 (38.01)
Are individuals living with dementia able to recognize when to use home safety interventions (e.g., holding handrails, using grab bars) without being reminded?	11 (9.09)	76 (62.81)	34 (28.09)
Are individuals living with dementia always able to utilize home safety interventions without being guided?	12 (9.92)	72 (59.50)	37 (30.58)
Participant response on digital intervention and readiness (N=121)			
Questions	Responses		
	Yes N (%)	Maybe N (%)	No N (%)
Do you think wearable electronic devices can help improve the safety of people living with dementia?	87 (71.90)	32 (26.45)	2 (1.65)
Do you think combining wearable electronic devices with existing home safety interventions would be advantageous for people living with dementia?	98 (80.99)	23 (19.00)	0 (0.00)
Would you be willing to take part in the assessment of the proposed system once it has been completed?	53 (43.80)	53 (43.80)	15 (12.40)
Do individuals living with dementia use any wearable electronic interventions or devices?	12 (9.92)	45 (37.19)	64 (52.89)
Do individuals living with dementia whom you care for or support use glasses?	59 (48.8)	38 (31.4)	24 (19.8)

### People with dementia assessment on independent mobility and home safety intervention

 Analysis of the independent mobility assessment contingency table (Table 3) revealed that only a few responses indicated a consistent ability among PwD across the various factors assessed. The majority of responses indicated that, despite the availability of home safety interventions, PwD are only sometimes (42.14%) able to maintain consistent awareness of their surroundings. Additionally, 29.75% of responses indicated a lack of awareness, while only 13.22% reported consistent awareness. Similarly, PwD were reported to sometimes navigate safely without assistance (39.67%), identify when to use safety interventions (50.41%), and utilize these interventions without guidance (47.11%). However, substantial proportions indicated difficulty in these areas, with 43 responses (35.54%), 34 (28.10%), and 37 (30.58%) respectively reporting inability to perform these tasks. 

**Table 3:** Independent mobility assessment contingency table and χ^2^ test.

Measures	Availability of home safety intervention		
	Yes	Sometimes	No
Consistently maintain awareness of surroundings			
Yes Sometimes No	16 (13.22%) 51 (42.14%) 36 (29.75%)	1 (0.83%) 14 (11.57%) 0 (0.00%)	1 (0.83%) 2 (1.65%) 0 (0.00%)
χ^2^=12.299; df=4; p=0.01526 Cramer’s V=0.2254			
Always able to navigate safely without assistance			
Yes Sometimes No	12 (9.92%) 48 (39.67%) 43 (35.54%)	0 (0.00%) 13 (10.74%) 2 (1.65%)	2 (1.65%) 0 (0.00%) 1 (0.83%)
χ^2^=18.125; df=4; p=0.001166 Cramer’s V=0.2737			
Able to know when to use safety interventions without reminder			
Yes Sometimes No	8 (6.61%) 61 (50.41%) 34 (28.10%)	1 (0.83) 14 (11.57%) 0 (0.00%)	2 (1.65%) 1 (0.83%) 0 (0.00%)
χ^2^=20.028; df=4; p=0.0004932 Cramer’s V=0.2877			
Able to know how to use safety intervention without guide			
Yes Sometimes No	9 (7.44%) 57 (47.11%) 37 (30.58%)	1 (0.83%) 14 (11.57%) 0 (0.00%)	2 (1.65%) 1 (0.83%) 0 (0.00%)
χ^2^=20.043; df=4; p=0.0004898 Cramer’s V=0.2878			

 According to Table 3, the Cramér’s V coefficients for each pair of variables ranged from 0.225 to 0.287, indicating a weak association between the variables. Despite the weak association, the low p-values (below 0.05) indicated statistically significant dependence. This suggests that the observed outcomes are unlikely to have occurred by chance. Therefore, there is sufficient evidence to reject the null hypothesis in favor of the alternative hypothesis. 

## DISCUSSION

 Previous studies recommend home modification strategies as a standard practice to enhance the safety of PwD at home^4,9,18,21^. Consistent with this, 78% of participants in the present study affirmed that home safety interventions to prevent falls are commonly installed in the residences of PwD. However, findings indicate that despite the presence of such interventions, PwD struggle to consistently maintain awareness of their surroundings. This results in low utilization of existing home safety measures, potentially undermining their intended purpose. Low adherence to these safety measures may be attributed to dementia-related symptoms, such as reduced spatial awareness and memory impairments, which significantly increase the risk of falls, particularly among individuals living alone. This observation aligns with existing research showing that PwD often have difficulty recognizing and responding to hazardous situations^30^. Similarly, PwD face challenges navigating stairs and uneven surfaces without assistance, identifying when to use safety interventions, and understanding how to use them independently. Even in environments designed to support independent living, additional support is often required to ensure safety^22^. 

 Given the substantial fall risk among PwD, especially those living alone, it is worth considering digital interventions as a complement to existing home safety measures to minimize fall hazards and related domestic accidents. This study suggests that digital interventions providing virtual cues related to home safety interventions may help PwD utilize them more effectively. Most participants supported this idea, believing such interventions could enhance PwD safety and express willingness to participate in testing and evaluating these solutions. Caregivers are increasingly interested in leveraging wearable and monitoring technologies to alleviate their workload and support older adults in maintaining independence at home^31^. The use of digital interventions can be challenging for individuals with cognitive impairments, and reluctance to wear devices poses an additional barrier, potentially resulting in low adoption rates^32,33^. The study affirmed that wearable electronic device usage among PwD remains low, with 52.89% of participants reporting no use and only 9.92% indicating some level of adoption. However, the use of prescribed traditional glasses is widespread, with 48.8% of participants reporting their usage, as also noted in related studies^34^. This study posits that designing wearable electronic devices in the form of glasses, leveraging users’ familiarity with them, could enhance comfort and acceptance. Transforming standard glasses frequently used by the aged^34^ into smart aids with a straightforward on/off feature may effectively overcome challenges related to technical acceptance and usability^33^. Smart glasses can enhance user experience by providing timely, contextual information about the surroundings. This capability is enabled by immersive technologies such as augmented reality (AR), which integrates virtual elements with the real-world environment. AR technologies surpass traditional visual-centric interfaces by incorporating audio and other non-visual cues, thereby unlocking new realms of interaction and engagement within an augmented environment^35^. Some studies^36^ have demonstrated the potential of AR-equipped smart glasses in mitigating domestic hazards by offering virtual cues and real-time feedback to users during dangerous situations. 

 In conclusion, this study found that digital interventions can support PwD in utilizing existing home safety interventions and navigating their environments more independently. Such interventions could help the target population understand when and how to use these resources, thereby enhancing the overall effectiveness of their implementation. 

### Strength and limitations

 This study introduced a new perspective by presenting evidence supporting the need for digital intervention for PwD, particularly those living alone, to effectively use existing home safety interventions and to move safely around their environments. The findings also reveal that PwD exhibits limited compliance with home safety interventions, largely due to the nature of dementia symptoms. Lack of adherence undermines the effectiveness of these interventions. While numerous studies have proposed strategies to enhance adherence to medication, exercise therapy, and other treatments for PwD^11,37-39^, there is no evidence suggesting that similar efforts are being made to improve adherence to home safety interventions. This study, therefore, highlights a gap for further research. 

 The study’s limitations include potential selection bias, as only caregivers’ perspectives were considered, excluding input from PwD. Additionally, the small and predominantly African ethnic sample may limit the generalizability of the findings. 

## Data Availability

The datasets generated and/or analyzed during the current study are not publicly available due to [ethical/legal/privacy] restrictions but are available from the corresponding author upon reasonable request.
